# Hematological Manifestations of COVID-19 and Their Prognostic Significance in an Intensive Care Unit: A Cross-Sectional Study

**DOI:** 10.7759/cureus.19887

**Published:** 2021-11-25

**Authors:** Nipun Bawiskar, Dhruv Talwar, Sourya Acharya, Sunil Kumar

**Affiliations:** 1 Department of Medicine, Jawaharlal Nehru Medical College, Datta Meghe Institute of Medical Sciences (Deemed to be University), Wardha, IND

**Keywords:** rt-pcr, sars-cov-2, cross-sectional study, hematological manifestations, covid-19

## Abstract

Introduction

Severe acute respiratory syndrome coronavirus 2 (SARS-CoV-2) started as a pathology chiefly affecting the lower respiratory tract. It was first encountered in Wuhan, China. As an infection with an unknown etiology, it was extensively studied in order to establish its profile with respect to the probable manifestations and required medical management. The hematological profile of a patient typically affected with coronavirus disease 2019 (COVID-19) showed lymphopenia with an altered neutrophil-lymphocyte ratio, raised inflammatory markers like D-dimer, interleukin 6 (IL-6), C-reactive protein (CRP), lactate dehydrogenase, and serum ferritin. The prognostic significance of these markers has been studied in this cross-sectional study.

Patients and methods

Data were collected from consecutive subjects admitted in the intensive care unit of Acharya Vinobha Bhave Rural Hospital, Sawangi Meghe, Wardha, who were aged more than 16 years and were later confirmed to be positive for COVID-19 through throat/nasal swab (rapid antigen test/reverse transcription-polymerase chain reaction (RT-PCR)). Written informed consent (by signature or thumbprint) was obtained from all participants. Statistical analysis was done by using descriptive and inferential statistics with the help of the chi-square test and z-test for the difference between two means. The software used in the analysis was SPSS 27.0 (IBM Corp., Armonk, NY) and GraphPad Prism 7.0 (GraphPad Software, San Diego, CA). P<0.05 was considered as the level of significance.

Results

A total of 200 patients were studied. Fifty-nine point five percent (59.5%) of those who succumbed were over 50 years of age and a significant number (23.5 %) had comorbidities like diabetes mellitus, hypertension, and chronic kidney disease. There was a significant positive correlation between the mortality rate and mean platelet volume (P=0.001), neutrophil-lymphocyte ratio (P=0.001), raised D-dimer (P=0.006), serum ferritin (P=0.0001), lactate dehydrogenase (P=0.0001), and C-reactive protein (P=0.0001).

Conclusion

The analysis of the data collected highlights the correlation between the studied hematological manifestations of COVID 19 and their association with the severity of the disease.

## Introduction

Coronavirus disease 2019 (COVID 19) is a pandemic that has crippled the world and has forced us to practice caution in terms of our interactions with people and our environment. It was first detected in China and is an enveloped, single-stranded RNA virus [1]. SARS-CoV-2 is about 79% identical to severe acute respiratory syndrome coronavirus 2 (SARS-CoV-2) and 50% identical to Middle East respiratory syndrome coronavirus (MERS-CoV). They are comparable at the nucleotide level, having a similar receptor-binding domain structure [2].

SARS-CoV-2 is transmitted from person to person by respiratory secretions such as droplets [3]. Upon exposure, the virus enters the host and binds to receptors. ACE2 or angiotensin-converting enzyme 2 is the main receptor and has a high affinity for the virus in comparison to CD209L, which is an alternate receptor with a much lower affinity [4]. ACE2 is expressed on the epithelium of the trachea, bronchial serous glands, bronchi, alveoli, and also on alveolar macrophages and monocytes. On entering these target cells, the virus beings its replication. These, in turn, release mature virions that infect new target cells [5]. ACE2 is a surface molecule and is expressed on the mucosal cells of the intestine, endothelial cells of arteries and veins, epithelial cells of renal tubules, immune cells, and cerebral neurons. This makes a wide range of cells vulnerable to the SARS-CoV-2 virus [6]. There are various hematological manifestations in COVID-19, lymphopenia being one of them. The development of atypical pneumonia with rapid deterioration is seen as a result of increased levels of proinflammatory cytokines termed cytokine release syndrome(CRS). Elevated chemokines and cytokines are linked with a higher number of neutrophils and monocytes in both peripheral blood and lungs hence implicating their role in lung pathology. CRS is thus seen as a major cause of morbidity and mortality in patients infected with SARS-CoV-2 [7].

Severe COVID-19 is characterized by increased blood concentrations of the cytokine interleukin-6 (IL-6) and other inflammatory cytokines. Elevated serum IL-6 is linked to respiratory failure, acute respiratory distress syndrome (ARDS), and poor clinical outcomes. It also regulates the expression of C-reactive protein (CRP), which is another biomarker of severity. A high concentration of Interleukin-6 binds to sIL-6R (soluble IL-6R), making a complex with a gp130 dimer on potentially all cell surfaces [6-7]. Because endothelial cells do not express mIL-6R, IL-6-sIL-6R-JAK-STAT3 signaling is initiated. This results in the production of VEGF, IL-8, MCP-1, and additional IL-6, plus reduced E-cadherin expression on the cells of the endothelium, resulting in a systemic "cytokine storm" [6-7]. Vascular endothelial growth factor (VEGF) and decreased E-cadherin expression cause vascular permeability and leakage, which cause hypotension and pulmonary dysfunction in ARDS [8].

NLR or neutrophil-to-lymphocyte ratio has been identified as an independent risk factor in the severe form of the disease. Patients with age ≥ 50 and NLR ≥ 3.13 were found to have a significant risk of developing severe ARDS [9].

About 36% of the cases studied in China were found to have raised D-dimer levels. Elevated D-dimer levels with disseminated intravascular coagulation are findings consistent with poor outcomes and significant mortality in patients with COVID-19 [10].

Chinese data are indicative of higher mortality rates and severe COVID-19-associated illness in an older age group and those with a serious underlying health condition. Most of the cases in China were mild, with deaths occurring in approx. 80% of those aged ≥60 years [11].

As mentioned above, multiple hematological parameters are affected in COVID-19, hence we conducted this study with the aim to correlate them with the outcome of patients admitted to a COVID-19 intensive care unit of rural central India.

## Materials and methods

Study design

This cross-sectional study was done in Acharya Vinobha Bhave Rural Hospital, Sawangi Meghe, by the department of medicine among COVID-19 patients from November 2020 to January 2021. All consecutive patients admitted in the intensive care unit, with age more than 16 years with a positive throat/nasal swab (reverse transcription-polymerase chain reaction (RT-PCR) or rapid antigen test) were enrolled for the study. Patients with hematological malignancies, coagulation disorders, and postoperative patients were excluded.

Laboratory evaluation

Throat, as well as nasal swabs, were obtained in order to diagnose COVID-19 using RT-PCR. These were processed in the Central Clinical Laboratory (CCL) located in Jawaharlal Nehru Medical College. This laboratory is recognized by the Indian Council of Medical Research (ICMR) for the same. The machine used for testing is called RT-PCR Quant Studio 5 (Applied Biosystems, Waltham, MA), which works on the principle of reverse transcription of ribonucleic acid to deoxyribonucleic acid for amplification. The kit used is the Meril COVID-19 One-Step RT-PCR kit (Meril, Chala, Vapi, India), which contains COVID-19 enzyme mix (lyophilized), COVID-19 primer-probe mix, enzyme mix buffer, COVID-19 PCR positive control, and COVID-19 negative control.

Hemogram was obtained using an automated machine - Pentra XLR Coulter Counter [Five Parts] (Horiba Medical, Montpellier, France) with a peripheral venous sample obtained on admission. NLR was calculated as the ratio of absolute neutrophil count (ANC) and absolute lymphocyte counts (ALC). These were calculated manually on peripheral smear.

Platelet indices were obtained using the same automated hematology analyzer. The parameters observed were platelet count, mean platelet volume, and platelet crit.

Interleukin 6 levels were measured through enzyme-linked immunosorbent assay (ELISA) using Robonic Readwell Touch ELISA Plate Analyser (Robonic (India) Pvt. Ltd., Thane, India) and Robonic Washwell Plate ELISA. The Interleukin 6 human ELISA kit was used. Sensitivity was less than 1 pg/ml.

Serum ferritin levels were measured by electrochemiluminescence immunoassay using the VITROS 5600 integrated system (Ortho Clinical Diagnostics, Mumbai, India). The kit consisted of Elecsys Ferritin (Roche Diagnostics, Basel, Switzerland), ready-to-use ferritin reagent, and CalSet.

Serum lactate dehydrogenase was quantified by the lactate kinetic method, serum C-reactive protein was determined by immunoturbidimetry, and D-dimer was appraised by an automated latex enhanced immunoassay. 

Ethical consideration

Ethical committee approval was taken prior to starting the study (Ref no: Datta Meghe Institute of Medical Science (Deemed to be University)/Institutional Ethical Committee/2020-21/8813 [DMIMS(DU)/IEC/2020-21/8813].

Statistical analysis

Statistical analysis was done by using descriptive and inferential statistics with the help of the chi-square test and z-test for the difference between two means. The software used in the analysis was SPSS 27.0 (IBM Corp, Armonk, NY) and GraphPad Prism 7.0 (GraphPad Software, San Diego, CA). P<0.05 was considered the level of significance.

## Results

A total of 200 patients admitted to the intensive care unit were enrolled for this study. Out of these patients, 81 patients had an age of less than 50 years, whereas 119 patients had an age of more than 50 years. A total of 125 (62.50%) patients were male and 75 patients (37.50%) were females.

Age was found to be correlated significantly with the outcome with a P-value of .0001. Mortality was higher in the age group above 50 years (80.77%) when compared to the age group less than 50 years (19.23%) as shown in Table 1. Gender, however, was not associated with outcome significantly.

**Table 1:** Correlation of age and gender with outcomes

		Outcome		Total	א2-value
		Discharge	Death		
Age in yrs	≤ 50 yrs	71(47.97%)	10(19.23%)	81(40.50%)	13.91 P=0.0001,S
	>50 yrs	77(52.03%)	42(80.77%)	119(59.50%)	
Total		148(100%)	52(100%)	200(100%)	
		Outcome		Total	א2-value
		Discharge	Death		
Gender	Male	91(61.48%)	34(65.38%)	125(62.50%)	0.24 P=0.61,NS
	Female	57(38.51%)	18(34.62%)	75(37.50%)	
Total		148(100%)	52(100%)	200(100%)	

Co-morbidities, including diabetes mellitus, hypertension, chronic kidney disease, and others (thyroid disorder, bronchial asthma, and coronary artery disease) were found to be correlated significantly with the outcome as shown in Table 2.

**Table 2:** Correlation of comorbidities with outcomes Others: Thyroid Disorder, Bronchial Asthma, Chronic Obstructive Pulmonary Disease

Comorbidity	Discharge	Death	Total	א2-value	p-value
Diabetes Mellitus	40(27.02%)	8(15.38%)	48(24%)	4.34	0.037,S
Hypertension	12(12.16%)	2(3.85%)	14(7%)	4.34	0.037,S
Chronic Kidney Disease	18(12.16%)	4(3.84%)	22(11%)	4.34	0.037,S
Others	1(0.68%)	0(0%)	1(0.50%)	0.35	0.55,NS

As shown in Table 3, interleukin 6 was correlated significantly with the outcome, with a p-value of 0.0001. Mean interleukin 6 in discharged patients was 247.03, whereas it was 658.21 in expired patients. Hematological parameters, including platelet count, mean platelet volume, and plateletcrit, were correlated with outcomes significantly. Inflammatory markers, including neutrophil-lymphocyte ratio, D-dimer, C-reactive protein, and lactate dehydrogenase, were correlated significantly with the outcome. Ferritin was also found to be correlated significantly with the outcome with a p-value of .0001. However, erythrocyte sedimentation rate and serum uric acid were not found to be significantly associated with outcomes.

**Table 3:** Showing correlation of different parameters with outcomes

Interleukin-6					
Outcome	N	Mean	Standard Deviation	Standard Error Mean	z-value
Discharge	148	4.95	1.87	0.15	31.12 P=0.0001,S
Death	52	50.13	17.45	2.42	
Platelet count					
Outcome	N	Mean	Standard Deviation	Standard Error Mean	z-value
Discharge	148	2.01	0.84	0.06	0.56 P=0.57,NS
Death	52	1.92	1.08	0.15	
Mean platelet volume					
Outcome	N	Mean	Standard Deviation	Standard Error Mean	z-value
Discharge	148	8.61	0.79	0.06	3.30 P=0.001,S
Death	52	9.03	0.8	0.11	
Plateletcrit					
Outcome	N	Mean	Std. Deviation	Standard Error Mean	z-value
Discharge	148	1.71	0.71	0.05	0.01 P=0.99,NS
Death	52	1.71	0.91	0.12	
Neutrophil-lymphocyte ratio					
Outcome	N	Mean	Std. Deviation	Std. Error Mean	z-value
Discharge	148	5.38	2.77	0.22	3.39 P=0.001,S
Death	52	6.88	2.63	0.36	
D-dimer					
Outcome	N	Mean	Std. Deviation	Std. Error Mean	z-value
Discharge	148	1.07	1.28	0.1	2.82 P=0.006,S
Death	52	1.95	2.13	0.29	
Lactate dehydrogenase					
Outcome	N	Mean	Std. Deviation	Std. Error Mean	z-value
Discharge	148	439.97	193.08	15.92	5.40 P=0.0001,S
Death	52	701.67	329.46	45.68	
C-reactive protein					
Outcome	N	Mean	Std. Deviation	Std. Error Mean	z-value
Discharge	148	8.51	7.2	0.59	4.94 P=0.0001,S
Death	52	15.09	8.58	1.19	
Serum ferritin					
Outcome	N	Mean	Std. Deviation	Std. Error Mean	z-value
Discharge	148	402.26	325.87	26.78	3.96 P=0.0001,S
Death	52	628.93	364.5	50.54	
Erythrocyte sedimentation rate					
Outcome	N	Mean	Standard Deviation	Standard Error Mean	z-value
Discharge	148	50.35	21.12	1.73	1.31 P=0.19,NS
Death	52	55	24.26	3.36	
Uric acid					
Outcome	N	Mean	Standard Deviation	Standard Error Mean	z-value
Discharge	148	5.04	2.09	0.17	0.42 P=0.69,NS
Death	52	5.19	2.4	0.33	

## Discussion

The COVID-19 pandemic has led to significant morbidity and mortality worldwide. It has an incubation period of up to two weeks. Patients present with non-specific symptoms and a picture that suggests hyperactivity, followed by CD8 and T cell depletion in the early stages of the illness [12]. Tang et al. studied ICU patients admitted in Singapore and found that the prognosis was worse in patients whose blood reports showed lymphopenia [13]. Deng et al. and Qin et al. reported similar findings in studies conducted by them [14-15]. A total of 187 patients were observed by Guo et al. with lymphopenia, altered NLR, and raised troponin as significant markers of severity in his study [16]. During the course of hospital stay, the patients analyzed by us had less than normal lymphocytes in addition to an increase in the neutrophil-lymphocyte ratio predominantly among the deceased.

In a meta-analysis of 9 studies, thrombocytopenia was present in those who succumbed to the disease [17]. In comparison, a peak in the Platelet counts was noted by Qu et al in critically ill patients observed by him [18]. Our study noted that although platelet counts were not of significance, the mean platelet volume was a good predictor of the severity of the disease with a p-value of 0.001.

Raised LDH was noted in 41% of the cases while raised CRP was found in 60.7% of the total cases studied by Guan et al. [19]. An increase in the levels of serum ferritin, IL-6, and LDH was observed by Zhou et al. among the 191 subjects studied by him and his colleagues [20]. Similar findings were noted by Wu et al.; he compared serum ferritin levels in relation to the severity in ARDS [21]. Our findings were consistent with those observed in these studies with the serum ferritin, LDH, and CRP being of paramount importance in assessing the prognosis of patients with COVID-19.

In a retrospective study done in China, a sequential increase in D-dimer was noted among patients who perished [14]. This was also observed by Tang et al. in a study done by him on 183 patients infected by SARS-CoV-2 [13]. Several studies concluded that immune deregulation and endothelial dysfunction played a part in the pathophysiology of COVID-19 and its systemic manifestations [20]. According to existing data, IL-6 surpasses CRP and other inflammatory indicators in predicting respiratory failure in COVID-19 [22]. Many studies suggest an increase in IL-6 levels in more severe cases [23]. These findings were substantiated as a considerable number of patients that succumbed in our cross-sectional analysis had a significant rise in IL-6 levels during the course of their hospital stay.

Thrombocytopenia linked with COVID-19 has a number of factors that might be contributory. There is a significant homology in the sequence of nucleotides seen in SARS-CoV-2 and other coronaviruses, including SARS-COV-2 and human coronavirus 229E (HCoV-229E) [24].

HCoV-229E is known to bind to the cells inside the bone marrow through CD 14 receptors, including platelets, monocytes, and granulocytes. This CD 13 receptor is also expressed in abundance in the respiratory tract and gastrointestinal tract inside the epithelial cells. The binding of HCoV-229E to the CD 13 receptor leads to uncontrolled growth followed by apoptosis in the bone marrow.

Another factor responsible for thrombocytopenia in COVID-19 is the rise in platelet destruction that occurs due to autoantibody formation resembling immune-mediated thrombocytopenia. This phenomenon was first reported with human immune deficiency virus 1 infected patients. In the bloodstream of infected patients, there are epitopes of the virus, which have the potential to mimic certain antigens which are otherwise found on the surface of the platelet. This leads to the formation of an antigen-antibody complex further processed by the complement system, causing the destruction of platelets [25].

In SARS patients, disseminated intravascular coagulation is a commonly encountered complication. The use of mechanical ventilation and systemic inflammatory response leads to damage to the endothelial cells, causing the activation of platelets and thrombosis, ultimately resulting in the consumption of platelets in a disseminated manner.

Lymphopenia is another important hematological manifestation seen in COVID-19. It is proposed that lymphopenia occurs as a result of cell membrane lysis and the cytotoxic effects exerted on lymphocytes by SARS-CoV-2 due to the expression of angiotensin convertase enzyme 2 receptor over the surface of lymphocytes. Xu et al. had demonstrated in a study that angiotensin convertase enzyme 2 receptor, which was earlier thought to be only present on the epithelial cells was also present on the infiltrating lymphocytes found in lungs, oral mucosa, digestive system, and various other organs [26]. Another postulated mechanism is the presence of lactic acidosis in critically ill patients, which can lead to an inhibitory effect on the lymphocytes [27].

In cases of severe COVID-19, some studies have found raised levels of lactic acid, which might explain the cause of lymphopenia in COVID-19 [28].

Suggestions

For further studies, we would suggest that the correlation of the severity of COVID-19 be checked with hematological parameters, which are often neglected. Also, as this study was based in an intensive care unit, we would suggest further studies to be conducted in stable COVID-19 patients and in patients with re-infection with COVID-19 to look for the changes in hematological parameters in them.

Limitations

Our study is a single-center study, which is the main limitation. Also, as our study was conducted in a rural hospital in central India, we could not perform serial monitoring of all the parameters, which have been studied due to financial constraints. As the study was conducted in an intensive care unit, the category of patients could not be divided into mild, moderate, and severe COVID-19 categories and correlation could only be studied with outcome in an intensive care unit and not with the severity of COVID-19. The death rate was also significantly raised (25%) in our study, as only patients who were admitted to the intensive care unit were enrolled.

## Conclusions

After assessing the data from 200 patients with COVID-19 we observed that people >50 years of age were more prone to develop a severe form of the disease. Mortality was more among patients with comorbidities like diabetes, hypertension, and chronic kidney disease playing a part in the fatal outcome. Raised neutrophil-lymphocyte ratio, mean platelet volume, IL-6, D-dimer, serum ferritin, LDH, and CRP were important prognostic markers in determining the severity of COVID-19.
